# Disease status, reasons for discontinuation and adverse events in 1038 Italian children with juvenile idiopathic arthritis treated with etanercept

**DOI:** 10.1186/s12969-016-0126-0

**Published:** 2016-12-20

**Authors:** Sara Verazza, Sergio Davì, Alessandro Consolaro, Francesca Bovis, Antonella Insalaco, Silvia Magni-Manzoni, Rebecca Nicolai, Denise Pires Marafon, Fabrizio De Benedetti, Valeria Gerloni, Irene Pontikaki, Francesca Rovelli, Rolando Cimaz, Achille Marino, Francesco Zulian, Giorgia Martini, Serena Pastore, Chiara Sandrin, Fabrizia Corona, Marta Torcoletti, Giovanni Conti, Claudia Fede, Patrizia Barone, Marco Cattalini, Elisabetta Cortis, Luciana Breda, Alma Nunzia Olivieri, Adele Civino, Rosanna Podda, Donato Rigante, Francesco La Torre, Gianfranco D’Angelo, Mauro Jorini, Romina Gallizzi, Maria Cristina Maggio, Rita Consolini, Alessandro De Fanti, Valentina Muratore, Maria Giannina Alpigiani, Nicolino Ruperto, Alberto Martini, Angelo Ravelli, on behalf of the Italian Pediatric Rheumatology Study Group

**Affiliations:** 1 Università degli Studi di Genova, Genova, Italy; 2 Istituto Giannina Gaslini, Genova, Italy; 3 Ospedale Pediatrico Bambino Gesù, Roma, Italy; 4 Istituto Ortopedico Gaetano Pini, Milano, Italy; 5 Ospedale Pediatrico Meyer, Firenze, Italy; 6 Università degli Studi, Padova, Italy; 7 IRCCS Burlo Garofolo, Trieste, Italy; 8 Fondazione IRCCS Cà Granda – Ospedale Maggiore Policlinico, Milano, Italy; 9 Azienda Ospedaliera Universitaria Policlinico G. Martino, Messina, Italy; 10 Azienda Ospedaliero-Universitaria Policlinico Vittorio Emanuele, Catania, Italy; 11 Università degli Studi di Brescia, Brescia, Italy; 12 Ospedale di Orvieto, Orvieto, Italy; 13 Università degli Studi di Chieti, Chieti, Italy; 14 Seconda Università degli Studi di Napoli, Napoli, Italy; 15 Azienda Ospedaliera Card. G. Panico, Tricase, Italy; 16 Ospedale Regionale per le Microcitemie, Cagliari, Italy; 17 Policlinico Universitario A. Gemelli, Roma, Italy; 18 Ospedale A. Perrino, Brindisi, Italy; 19 Università Politecnica delle Marche, Ancona, Italy; 20 Ospedale Pediatrico G. Salesi, Ancona, Italy; 21 Università degli Studi di Messina, Messina, Italy; 22 Ospedale dei Bambini G. Di Cristina, Palermo, Italy; 23 Università degli Studi di Pisa, Pisa, Italy; 24 Arcispedale S.Maria Nuova, Reggio Emilia, Italy; 25 IRCCS Fondazione Policlinico San Matteo, Pavia, Italy; 26 Pediatria II-Reumatologia, Istituto G. Gaslini, Largo G. Gaslini 5, 16147 Genova, Italy

**Keywords:** Juvenile idiopathic arthritis, Etanercept, Pediatric rheumatology, TNF inhibitors, Biologic therapies

## Abstract

**Background:**

Data from routine clinical practice are needed to further define the efficacy and safety of biologic medications in children with juvenile idiopathic arthritis (JIA). The aim of this analysis was to investigate the disease status, reasons for discontinuation and adverse events in Italian JIA patients treated with etanercept (ETN).

**Methods:**

In 2013, all centers of the Italian Pediatric Rheumatology Study Group were asked to make a census of patients given ETN after January 2000. Patients were classified in three groups: group 1 = patients still taking ETN; group 2 = patients discontinued from ETN for any reasons; group 3 = patients lost to follow-up while receiving ETN. All three groups received a retrospective assessment; patients in group 1 also underwent a cross-sectional assessment.

**Results:**

1038 patients were enrolled by 23 centers: 422 (40.7%) were in group 1, 462 (44.5%) in group 2, and 154 (14.8%) in group 3. Median duration of ETN therapy was 2.5 years. At cross-sectional assessment, 41.8% to 48.6% of patients in group 1 met formal criteria for inactive disease, whereas 52.4% of patients in group 2 and 55.8% of patients in group 3 were judged in clinical remission by their caring physician at last visit. A relatively greater proportion of patients with systemic arthritis were discontinued or lost to follow-up. Parent evaluations at cross-sectional visit in group 1 showed that 52.4% of patients had normal physical function, very few had impairment in quality of life, 51.2% had no pain, 76% had no morning stiffness, and 82.7% of parents were satisfied with their child’s illness outcome. Clinically significant adverse events were reported for 27.8% of patients and ETN was discontinued for side effects in 9.5%. The most common adverse events were new onset or recurrent uveitis (10.2%), infections (6.6%), injection site reactions (4.4%), and neuropsychiatric (3.1%), gastrointestinal (2.4%), and hematological disorders (2.1%). Ten patients developed an inflammatory bowel disease and 2 had a malignancy. One patient died of a fulminant streptococcal sepsis.

**Conclusions:**

Around half of the patients achieved complete disease quiescence under treatment with ETN. The medication was overall well tolerated, as only one quarter of patients experienced clinically significant adverse events and less than 10% had treatment discontinued for toxicity.

## Background

Etanercept (ETN), a tumor necrosis factor (TNF) antagonist, has been the first biologic agent registered for use in children with juvenile idiopathic arthritis (JIA). Its efficacy and safety have been established in a randomized placebo-controlled withdrawal trial in patients with a polyarticular disease course who were refractory or intolerant to methotrexate (MTX) [1]. Long-term extension studies of the original trial cohort and national registries have subsequently confirmed the sustained clinical benefit and acceptable tolerability of the drug [2–5]. The evidence for the effectiveness of ETN in JIA has been expanded by the observation that its administration is associated with improvement of functional ability and health-related quality of life [6–8], recovery of growth velocity and bone status [9, 10], and inhibition of progression of radiographic joint damage [11]. Recent studies have shown that around half of children with JIA who are treated with ETN in clinical practice are able to attain complete disease quiescence [12–14]. However, there is a need of more data from large series of patients treated in real-life clinical settings to fully characterize the efficacy and safety profile of this medication.

In June 2013, the Italian Pediatric Rheumatology Study Group undertook a national multicenter survey aimed to investigate the disease status, reasons for discontinuation and adverse events in Italian patients with JIA who were receiving or had received ETN. The findings of this study, named Etanercept in Italian Children with Arthritis (EtICA) study, are described in the present article.

## Methods

### Study design and patient enrolment

All centers that are part of the Italian Pediatric Rheumatology Study Group were invited to participate in this multicenter, observational study. To minimize a selection bias, investigators at each center were first asked to make a census of all patients with JIA by the International League of Associations for Rheumatology (ILAR) criteria [15] who were given ETN at the center after January 2000 and had received the medication for at least 6 months. Based on the status of ETN therapy at the time of the census, patients were classified in three groups: group 1 = patients still taking ETN; group 2 patients discontinued from ETN for any reasons; group 3 patients lost to follow-up while receiving ETN. Next, each investigator was asked to make a retrospective assessment for all patients in groups 1, 2 and 3, and a cross-sectional assessment at first visit after the census for all patients in group 1. Study data were collected in ad hoc case reports forms. Once completed, all forms were sent to the coordinating center (the Gaslini Institute, Genoa, Italy). Ethics committee approval of the study protocol was obtained in all participating centers.

### Retrospective assessment

The following information was collected through the review of patients charts: sex, age at disease onset, ILAR category, disease duration at first visit at study center and at start of ETN, medications administered before ETN start and during ETN therapy, ETN-related side effects, duration of ETN therapy at treatment discontinuation (for patients in group 2) or at last follow-up visit (for patients in group 3), reasons for ETN discontinuation (for patients in group 2), and disease activity state (remission or active disease) at last follow-up visit (for patients in group 3).

### Cross-sectional assessment

The following clinical evaluations were performed at cross-sectional visit in patients still taking ETN (group 1). The caring physician made a standardized joint examination and recorded the count of joints with swelling, pain/tenderness, restricted motion, and active arthritis [16]; in addition, he or she also rated the overall disease activity on a 21-numbered circle visual analog scale (VAS; where 0 = no activity and 10 = maximum activity) [17]. The same physician also assessed the amount of articular and extra-articular damage through the Juvenile Arthritis Damage Index (JADI) [18]. Briefly, the JADI-Articular (JADI-A) assesses 36 joints or joint groups for the presence of damage, and the damage observed in each joint is scored on a 3-point scale (0 = no damage, 1 = partial damage, and 2 = severe damage, ankylosis, or prosthesis). The maximum total score is 72. The JADI-Extraarticular (JADI-E) includes 13 items in 5 different organs/systems. Each item is scored as 0 if damage is absent or as 1 if damage is present. Due to the relevant impact of ocular damage on the child’s health, in each eye a score of 2 is given in case the patient has had ocular surgery, and a score of 3 is given in case the patient has developed legal blindness. The maximum total score is 17.

Prior to the physician visit, a parent (or guardian) completed the parent version of the Juvenile Arthritis Multidimensional Questionnaire (JAMAR) [19]. This questionnaire incorporates all main parent-centered outcome measures, including physical function, health-related quality of life (HRQL), overall well-being, pain, level of disease activity, self- or proxy-reported joint count, morning stiffness, assessment of disease status and course, side effects of medications, therapeutic compliance, difficulties at school, and satisfaction with illness outcome. We previously found that the mean (SD) HRQL score in 801 healthy children was 2.6 (2.4) and that the mean (SD) score of the physical health and psychosocial health subscales was 0.8 (1.2) and 1.8 (1.7), respectively (Bertamino M et al. Unpublished observation).

Laboratory investigations included the erythrocyte sedimentation rate (ESR) and the C-reactive protein (CRP), but were performed only if deemed necessary by the caring physician for clinical purposes.

Informed consent to participate in the study was provided by both the parent/guardian and the patient (when applicable) for all patients who underwent the cross-sectional assessment.

### Definitions of disease activity states

In patients still taking ETN (group 1), the state of disease activity at cross-sectional visit was assessed through the following criteria: 1) criteria for inactive disease (ID) in JIA [20]; 2) preliminary definition of minimal disease activity in JIA [21]; 3) criteria for inactive disease and low disease activity (LDA), moderate disease activity (MDA), and high disease activity (HDA) in JIA based on the Juvenile Arthritis Disease Activity Score (JADAS) and the clinical JADAS (cJADAS) [22–24]. To harmonize the terminology of disease activity states with that used in the adult rheumatology community, in this study, the term minimal disease activity has been replaced with LDA, which has an identical meaning. For patients in groups 2 and 3, the disease activity state at treatment discontinuation or last follow-up visit, respectively, was defined by the caring physician based on the data recorded in the clinical chart.

### Recording of adverse events

ETN-related adverse events were recorded for all patients in groups 1, 2 and 3. Study investigators were instructed to report all adverse events that they considered clinically significant, without pre-specified definitions of characteristics and severity. Adverse events that led to treatment discontinuation were registered for patients who had ETN discontinued for side effects.

### Statistics

Descriptive statistics are reported as median and interquartile range (IQR) for continuous variables and as absolute frequency and percentage for categorical variables. Comparisons of disease characteristics between patient groups were performed by means of Mann-Whitney U test in case of quantitative data and by means of the chi-square test, or the Fisher’s Exact test, as appropriate, in case of categorical data. All statistical tests were two-sided; a *P* value of less than 0.05 was considered as statistically significant. The statistical package used were SAS 9.3 (Institute Inc., Cary, NC, USA) and Statistica (version 8.0, StatSoft Corp., Tulsa, OK).

## Results

All 26 invited pediatric rheumatology centers agreed to participate in the study and included in the census a total of 1231 patients. However, 3 centers, which had listed 109 patients, did not provide any evaluations and were then excluded. Of the remaining 1122 patients, 1068 (95.2%) underwent the study assessments. Thirty of them were enrolled by more than one center and were, thus, counted only once (by retaining the most recent assessment), leading to a final number of assessed patients of 1038. Of them, 422 (40.7%) were still receiving ETN (group 1), 462 (44.5%) had been discontinued from ETN (group 2), and 154 (14.8%) had been lost to follow-up while receiving ETN (group 3). For the purpose of the analyses, patients in group 1 were placed in a “cross-sectional group” and patients in groups 2 and 3, who received only retrospective assessments, were combined in a single “retrospective group”.

The characteristics of the study patients at the start of ETN therapy are presented in Table 1. Overall, the study cohort was characterized by marked female prevalence, young age at onset, and high frequency of ANA positivity. The median disease duration (3.5 years) was quite long, but the average duration of follow-up at the study center was 2.1 years. Around two third of patients had extended oligoarthritis or rheumatoid factor-negative polyarthritis. Patients with persistent oligoarthritis outnumbered those with psoriatic arthritis, enthesitis-related arthritis, or undifferentiated arthritis. There were more patients with systemic arthritis in the retrospective group than in the cross-sectional group. Patients in the cross-sectional group had a younger age at disease onset and were more frequently ANA positive than those in the retrospective group. Before the start of ETN, approximately 90% of the patients had received MTX, 50% systemic corticosteroids, and 55% intra-articular corticosteroids. 53 patients were given biologic medications other than ETN before treatment start, which included adalimumab (*n* = 20), infliximab (*n* = 19), anakinra (*n* = 15), abatacept (*n* = 3), and tocilizumab (*n* = 1). During ETN therapy, more than 70% of the patients were given concomitant MTX therapy and around 25% corticosteroids, either systemic or intra-articular. The median (IQR) duration of ETN therapy in the cross-sectional and retrospective group was 2.5 (1–4.2) years and 2.6 (1.3–4.4) years, respectively. 79.5% of patients were treated for at least 1 year, 58.5% for at least 2 years, and 19.1% for at least 5 years.

**Table 1:** Baseline characteristics of children with juvenile idiopathic arthritis treated with etanercept^a^

	All patients (*n* = 1038)	Cross-sectional group^b^ (*n* = 422)	Retrospective group^c^ (*n* = 616)	*P*
Female	781 (75.2)	335 (79.4)	446 (72.4)	0.01
ILAR category				
Systemic arthritis	106 (10.2)	21 (5)	85 (13.8)	<0.0001
Persistent oligoarthritis	139 (13.4)	60 (14.2)	79 (12.8)	0.52
Extended oligoarthritis	325 (31.3)	147 (34.8)	178 (28.9)	0.04
RF-negative polyarthritis	329 (31.7)	138 (32.7)	191 (31)	0.56
RF-positive polyarthritis	50 (4.8)	16 (3.8)	34 (5.5)	0.20
Enthesitis-related arthritis	48 (4.6)	21 (5)	27 (4.4)	0.65
Psoriatic arthritis	34 (3.3)	16 (3.8)	18 (2.9)	0.44
Undifferentiated arthritis	7 (0.7)	3 (0.7)	4 (0.6)	1.0
Patients with positive ANA	586/1028 (57)	278 (66.5)	308 (50.5)	<0.0001
Median (IQR) age at disease onset, years	4.1 (2–8.3)	3.5 (1.8–7.3)	4.7 (2.1–9.1)	0.001
Median (IQR) age, years	10.1 (6.2–13.9)	9.6 (5.8–13.6)	10.4 (6.9–14.2)	0.04
Median (IQR) disease duration, years	3.5 (1.3–7.5)	3.6 (1.4–7.8)	3.3 (1.3–7.3)	0.16
Patients aged < 4 years	122 (11.8)	54 (12.8)	68 (11)	0.39
Median (IQR) follow-up duration^d^, years	2.1 (0.6–5.5)	2.4 (0.7–6.3)	2.1 (0.5–5.1)	0.04
Treatments before ETN start				
NSAIDs	603 (58.1)	269 (63.7)	334 (54.2)	0.002
Systemic corticosteroids	527 (50.8)	198 (46.9)	329 (53.4)	0.04
Intra-articular corticosteroids	579 (55.8)	263 (62.3)	316 (51.3)	0.0004
Methotrexate	930 (89.6)	387 (91.7)	543 (88.1)	0.07
Other synthetic DMARDs	187 (18)	48 (11.4)	139 (22.6)	<0.0001
Other biologic agents	53 (5.1)	18 (4.3)	35 (5.7)	0.31
Concomitant therapies during ETN administration				
Systemic corticosteroids	267 (25.7)	93 (22)	174 (28.2)	0.02
Intra-articular corticosteroids	257 (24.8)	98 (23.2)	159 (25.8)	0.34
Methotrexate	749 (72.2)	311 (73.7)	438 (71.1)	0.36
Other synthetic DMARDs	73 (7)	17 (4)	56 (9.1)	0.002

*ILAR* International League of Associations for Rheumatology, *ANA* antinuclear antibodies, *ETN* etanercept, *IQR* interquartile range, *NSAIDs* nonsteroidal anti-inflammatory drugs, *DMARDs* disease-modifying antirheumatic drugs

^a^Data are number (percentage) unless otherwise indicated

^b^Includes patients still receiving ETN

^c^Includes patients discontinued from ETN or lost to follow-up

^d^Duration of follow-up at study center

Table 2 shows the disease activity states and concomitant therapies at cross-sectional visit in patients still receiving ETN (cross-sectional group). The percentage of patients who met the Wallace criteria for ID [20] and the Magni-Manzoni criteria for LDA (or minimal disease activity) [21] was 41.8 and 63.6%, respectively. Notably, the exclusion of the criterion that requires the normality of acute phase reactants from Wallace criteria led to increase the proportion of patients with ID to 51%. The percentage of patients who met the JADAS10 criteria for ID, LDA, MDA and HDA [22, 23] was 46.4, 64.0, 24.4, and 11.6%, respectively. The figures obtained for the cJADAS10 [24] were similar. 68.5% of patients had no active joints and 56.6% had a physician global assessment indicating no disease activity. More than 80% of patients had normal ESR or CRP. 77.3 and 86.9% of patients had no evidence of articular or extra-articular damage, respectively. As compared to treatment baseline, the percentage of patients who were still receiving concomitant MTX therapy at cross-sectional visit was decreased from 73.7 to 47.4% and the percentage of patients who were taking systemic corticosteroids was diminished from 22 to 4.7%. Only 2.6% of patients underwent intra-articular corticosteroid administration at cross-sectional visit. Of the 21 patients with systemic arthritis who were still receiving ETN, 15.8 and 9.5% had ID by Wallace and JADAS criteria, respectively, and 23.8 and 28.6% had LDA by Magni-Manzoni and JADAS criteria, respectively.

**Table 2:** Therapeutic data and disease activity states at cross-sectional visit in patients still receiving etanercept (*n* = 422)^a^

	No assessed	No (%) positive
Concomitant therapies		
Systemic corticosteroids	422	20 (4.7)
Intra-articular corticosteroids	422	11 (2.6)
Methotrexate	422	200 (47.4)
Salazopyrin	422	3 (0.7)
Azathioprine	422	1 (0.2)
Disease activity states – Formal definitions		
Wallace criteria for inactive disease	392	164 (41.8)
Wallace criteria for inactive disease without acute phase reactants	420	214 (51)
Magni-Manzoni criteria for low disease activity	420	267 (63.6)
Disease activity states – JADAS10		
Inactive disease	422	196 (46.4)
Low disease activity^b^	422	270 (64)
Moderate disease activity	422	103 (24.4)
High disease activity	422	49 (11.6)
Disease activity states – cJADAS10		
Inactive disease	422	205 (48.6)
Low disease activity^b^	422	246 (58.3)
Moderate disease activity	422	117 (27.7)
High disease activity	422	59 (14)
Patients with no active joints	422	289 (68.5)
Patients with no swollen joints	422	313 (74.2)
Patients with no tender joints	422	299 (70.9)
Patients with no restricted joints	422	253 (60)
Patients with physician’s VAS = 0	422	239 (56.6)
Patients with ESR < 20 mm/h	398	321 (80.7)
Patients with normal CRP	402	341 (84.8)
Patients with JADI Articular = 0	419	324 (77.3)
Patients with JADI Extraarticular = 0	419	364 (86.9)

*IQR* interquartile range, *JADAS* Juvenile Arthritis Disease Activity Score, *VAS* visual analog scale, *ESR* erythrocyte sedimentation rate, *CRP* C-reactive protein, *JADI* Juvenile Arthritis Damage Index

^a^Data are number (percentage) unless otherwise indicated

^b^Includes patients with inactive disease

Parent-reported outcomes obtained at cross-sectional visit through the completion of the JAMAR are summarized in Table 3. Around half of the patients had normal physical function and only 22.1% had impairment in HRQL, as demonstrated by a HRQL score 1SD above the mean of healthy children. HRQL impairment was more common in the physical than in the psychosocial domains (48.7% vs. 19.7%). 46% of patients had a parent global assessment indicating good well-being and around half had no pain. The median (IQR) pain VAS score was 0 and, among patients who had a pain score > 0, 68.8, 23.6 and 7.6% had a score ≤ 1, 1–5 or > 5, respectively. Three quarter of patients had no morning stiffness and around two third were judged by their parents as being in the state of remission. 82.7% of parents were satisfied with the current state of their child’s illness.

**Table 3:** Parent-reported outcomes at cross-sectional visit in patients still receiving ETN

	No assessed	
Median (IQR) physical function score^a^	420	0 (0; 3)
No (%) of patients with physical function score = 0	420	220 (52.4)
Median (IQR) HRQL score^b^	417	2 (0; 5)
No (%) of patients with HRQL score > 1 DS above the mean of healthy children	417	92 (22.1)
Median (IQR) HRQL PhH score^c^	417	1 (0; 3)
No (%) of patients with HRQL PhH score > 1 DS above the mean of healthy children	417	203 (48.7)
Median (IQR) HRQL PsH score^c^	417	1 (0; 3)
No (%) of patients with HRQL PsH > 1 DS above the mean of healthy children	417	82 (19.7)
Median (IQR) VAS well-being^d^	420	0.5 (0; 3)
No (%) of patients with VAS well-being = 0	420	193 (46)
Median (IQR) VAS pain^d^	420	0 (0; 2,5)
No (%) of patients with VAS pain = 0	420	215 (51.2)
No (%) of patients with VAS pain ≤1	420	289 (68.8)
No (%) of patients with VAS pain from 1.5 to 5	420	99 (23.6)
No (%) of patients with VAS pain >5	420	32 (7.6)
Median (IQR) VAS disease activity^d^	420	0 (0; 2)
No (%) of patients with VAS disease activity = 0	420	224 (53.3)
No (%) of patients with no morning stiffness	420	319 (76)
Disease activity state		
No (%) of patients with remission	421	286 (67.9)
No (%) of patients with continued activity	421	86 (20.4)
No (%) of patients with disease flare	421	49 (11.6)
No (%) of parents satisfied with their child’s illness outcome	399	330 (82.7)

*IQR* interquartile range, *HRQL* health-related quality of life, *PhH* Physical Health, *PsH* Psychosocial Health, *VAS* visual analog scale

^a^Score ranges from 0 (no disability) to 30 (maximum disability)

^b^Score ranges from 0 to 30, higher scores indicate worse HRQL

^c^Score ranges 0–15, higher scores indicate worse HRQL

^d^All VAS range from 0 (best) to 10 (worst)

The achievement of disease remission was the leading cause of treatment discontinuation (52.4% of patients), followed by treatment inefficacy (29%), adverse events (21%), and parent decision/lack of adherence (2.6%). More than half of the patients lost to follow-up (55.8%) were in clinical remission at the time of last visit. Of the 70 patients with systemic arthritis who were discontinued from ETN, 19 (27.1%) and 45 (64.3%) had treatment withdrawn for disease remission and inefficacy, respectively.

Among the 108 patients (10.4%) who had not received MTX before ETN start, 35 were in the cross-sectional group and 73 were in the retrospective group. In the former group, the frequency of ID by Wallace and JADAS criteria was 25 and 28.6%, respectively, and the frequency of LDA by Magni-Manzoni and JADAS criteria was 44.1 and 51.4%, respectively. 71% per cent of the patients in the latter group had disease remission at treatment discontinuation or last follow-up visit.

One or more adverse events were reported for 27.8% of the patients. The most common were new onset or recurrence of uveitis (10.2%), infections (6.6%), injection site reactions (4.4%), neuropsychiatric disorders (3.1%), gastrointestinal disorders (2.4%), hematological disorders (2.1%), muco-cutaneous disorders (1.9%), and pain at the injection site (1.8%). Infectious complications were most frequently mild as in only 13 of 68 cases they led to treatment withdrawal. The side effects that led to treatment discontinuation in the 99 patients who were withdrawn from ETN for toxicity are presented in Table 4. Those that led to treatment discontinuation in more than 5% of patients were recurrence or new onset of uveitis, neuropsychiatric disorders, gastrointestinal disorders, infections, muco-cutaneous disorders, and hematologic disorders. Among these patients, the most common neuropsychiatric manifestations were behavioral disorders and headache, whereas urticaria and leukopenia were the most frequently reported mucocutaneous and hematologic disorders, respectively. Seven patients had injection site reaction or pain at injection site. Only 1 patient developed tuberculosis.

**Table 4:** Adverse events that led to treatment discontinuation in 99 patients

Adverse event	N
Recurrent or new-onset uveitis	38
Neuropsychiatric disorders	21
Behavioral disorders	7
Headache	6
Mood disorders/difficulty concentrating	4
Tics/unintentional movements	2
Papilledema	1
Hypoglossal nerve paralysis	1
Gastrointestinal disorders	15
Inflammatory bowel diseases	10
Persistent hypertransaminasemia	1
Abdominal pain	1
Peritonitis anti-DNA positive	1
Acute pancreatitis	1
Nausea or vomiting	1
Infection	13
Recurrent herpes labialis	2
Recurrent upper airway infections	2
Fatal streptococcal sepsis	1
Tuberculosis	1
Varicella complicated by porpora fulminans and fasciitis	1
Osteomyielitis	1
Cellulitis	1
Herpetic neuritis	1
Herpes zoster	1
CMV hepatitis	1
Recurrent bronchitis	1
Mucocutaneous disorders	10
Urticaria	4
Urticaria angioedema	2
Cutaneous vasculitis	2
Itch	1
Anal Condylomatosis	1
Haematological disorders	5
Leukopenia	3
Autoimmune thrombocytopenia	1
Hypocomplementemia	1
Injection site reactions	4
Pain at injection site	3
Malignancy	2
Thyroid carcinoma	1
Bladder carcinoma	1
Others	6
Persistent cervical lymphadenopathy	2
Autoimmune thyroiditis	1
Menometrorrhagia	1
Abortion	1
Renal lithiasis	1

The prevalence of uveitis was 52/314 (16.6%) among patients who were started with ETN before December 2006 and 54/724 (7.5%) among patients treated after January 2007. Furthermore, the 38 patients who had ETN discontinued due to the occurrence of uveitis had a median duration of ETN therapy longer than that of the entire patient cohort (3.4 and 2.5 years, respectively). Of the 10 patients who developed an inflammatory bowel disease, 4 had rheumatoid-factor negative polyarthritis, 2 oligoarthritis, 2 enthesitis-related arthritis, 1 rheumatoid-factor positive polyarthritis, and 1 psoriatic arthritis. Two patients had a malignancy while receiving ETN. The first was a 16-year old boy with persistent oligoarthritis who developed a thyroid carcinoma 1 year after the start of ETN. The disease duration at start of ETN was 3.7 years and the medication regimen before ETN initiation had included MTX and intra-articular corticosteroids. The second patient had a bladder carcinoma after 9.4 years of ETN administration. He was a boy with systemic arthritis who had a disease duration of 5.8 years at ETN start and had previously received MTX and systemic corticosteroids and intra-articular corticosteroids. The sole patient in the study cohort who died was a 5-year old child with systemic arthritis who was taking ETN and MTX and had a fulminant streptococcal sepsis, which developed as a complication of bilateral bronchopneumonia and pleurisy. He had previously received systemic corticosteroids and anakinra.

## Discussion

Our study describes the experience with the use of ETN in Italian children with JIA. Because all but 3 centers that are part of the Italian Pediatric Rheumatology Study Group participated in the study and more than 95% of patients included in the census underwent the cross-sectional or retrospective assessments, the results of our survey are generalizable to all JIA patients who received ETN in Italy in the study period. To obtain a precise and reliable documentation of the disease status of children on ongoing ETN therapy, we applied a comprehensive set of physician-centered and parent-centered outcome measures. These data provide a benchmarking for future outcome comparisons with other cohorts of JIA patients under therapy with ETN or other biologic medications.

We found that 41.8 to 48.6% of patients (depending of the definition used) who were still receiving ETN at the time of the census met the criteria for ID, 52.4% of patients who were discontinued from ETN had the medication stopped for clinical remission, and 55.8% of patients who were lost to follow-up were in clinical remission at last visit. Altogether, these findings concur with earlier reports that around half of JIA patients treated with ETN in standard clinical care are able to attain complete disease quiescence [12–14]. The strong disease-controlling potential of ETN was strengthened by the observation that in the cross-sectional sample 58.3 to 64% of patients had LDA and less than 15% had HDA. In addition, at cross-sectional visit the proportion of patients who were taking concomitant MTX or systemic corticosteroid therapy was decreased from 73.7 to 47.4% and from 22 to 4.7%, respectively, compared to treatment baseline. Only a small proportion of patients had evidence or articular or extra-articular damage.

These figures do not apply to the systemic arthritis subgroup, however, as there was a relatively greater proportion of patients with this JIA category among those who were discontinued or lost to follow-up than among those who were still taking ETN. A number of prior studies have shown that anti-TNF agents are less effective in the systemic subset of JIA [2, 3, 5, 12, 25–27]. This phenomenon has been attributed to interleukin (IL)-1 and IL-6 playing a greater pathogenetic role than TNF in systemic arthritis [28, 29]. However, a sizeable proportion of our patients with systemic arthritis had ID or LDA at cross-sectional visit or had ETN discontinued for the achievement of disease remission. Some studies have suggested that TNF inhibitors may be effective in the later afebrile disease phase of systemic JIA, characterized by chronic arthritis [4, 30].

The results of parent-centered assessments at cross-sectional visit corroborated the good disease status achieved by patients on continued ETN therapy, as 52.4% of them had normal physical function, a few had impairment in HRQL, 51.2% had no pain, 76% had no morning stiffness, and 67.9% were judged as having disease remission by the parent. In addition, 82.7% of parents were satisfied with their child’s illness outcome. That around half of the patients had persistent pain is worrying, however, although only 7.6% had severe pain (i.e.,a pain level > 5 on a 0-10 VAS). A recent study found that a subgroup of JIA patients treated with TNF inhibitors had continuous pain despite good disease control [31]. These observations underscore the possible persistence of clinically significant pain in children treated with biologic agents, even when good disease control is achieved. Constant monitoring of pain and its determinants remains, therefore, a clinical priority in the current biologic era [32]. Notably, use of nonsteroidal anti-inflammatory drugs before ETN start was reported for only 58% of patients. This finding underscores the recent change in treatment practice related to these medications.

ETN was generally well tolerated, as clinically significant adverse events were reported for 27.8% of patients and the medication was discontinued for side effects in 99 patients (9.5%). Overall, safety findings are consistent with those reported in other analyses of ETN in JIA [2, 4, 26, 33, 34].

New-onset or recurrent uveitis was the adverse event most commonly recorded in the entire study sample and was most frequently responsible for treatment discontinuation. This finding is important as ocular inflammation has been postulated to be related to ETN [35, 36]. It should be taken into account, however, that our sample included a high proportion of children with early-onset and ANA-positive disease, who have a high background risk of uveitis [37]. It has been hypothesized that ETN may not directly cause the development of uveitis, but the discontinuation of MTX upon successful arthritis control may pose the patient at risk [38]. Recent data have shown that MTX may prevent the onset of uveitis in children with JIA [39, 40]. Nevertheless, many patients who had ETN discontinued for uveitis were likely switched to adalimumab and infliximab, which are nowadays recommended as second-line therapeutic option among patients with JIA and uveitis [41]. Notably, the frequency of uveitis was higher among patients treated in the earlier years of ETN use than in those who received ETN in the recent years (16.6% versus 7.5%), which suggests that the increased awareness of the possible relation of ETN with ocular inflammation might have led to decrease its prevalence. In addition, the 38 patients who had ETN discontinued because of the occurrence of uveitis had an average treatment duration longer than that of the entire patient cohort.

Previous studies have shown that the risk of infection is increased in children with JIA receiving ETN as compared to those treated with MTX [4, 26, 30, 42, 43]. Infection was the second most prevalent adverse event in our cohort, but only ranked fourth among the causes of treatment discontinuation for side effects. Indeed, infectious complications were most frequently mild as in only 13 of 68 cases they led to treatment withdrawal. It is noteworthy that only 1 patient had tuberculosis. One patient, who was taking ETN and MTX, died for a fulminant streptococcal sepsis. A similar case has been reported previously [44].

Neuropsychiatric symptoms were the second most frequent cause of treatment discontinuation for adverse events. A high frequency of psychiatric symptoms was reported in other studies of ETN in JIA [2, 4]. It is, however, difficult to dissect the responsibility of the medication itself from behavioral or mood disorders related to psychological disease burden or due to other medications, namely MTX [45]. Unfortunately, the design of our study did not allow us to establish whether they were related to ETN by investigating whether they had disappeared after its withdrawal.

Ten patients developed an inflammatory bowel disease (IBD). Although this phenomenon has been observed in other studies [46, 47], the relationship between ETN and gut inflammation is unclear. It has been speculated that an already established subclinical IBD may be activated by the use of TNF inhibitors [48]. Along this line, it is conceivable that children with enthesitis related arthritis are more susceptible to this phenomenon, as IBD are closely associated with spondyloarthropathies [49]. However, only 2 of the 10 patients who developed IBD in our cohort had this JIA category. The role of ETN in inducing IBD deserves further investigations.

In spite of earlier alarming data [50], there is currently no evidence of an increased incidence of malignancy among patients exposed to TNF inhibitors compared with MTX [36], and the increased risk of malignancy has been attributed to the autoimmune disease itself, rather than to treatment with TNF inhibitors [51–53]. Two malignancies were observed in our cohort, one thyroid carcinoma and one bladder carcinoma. This frequency is in the range of that observed in other studies of similar size [38, 54]. These reports underscore the need to follow carefully JIA patients exposed to biologics and to continue long-term observation in adulthood. Further knowledge about risk profiles of these medications will be gained with for the accumulation of long-term data through national and international registries [55]. In 2011, the Pediatric Rheumatology INternational Trials Organisation (PRINTO) and the Pediatric Rheumatology European Society (PRES) created an international observational registry aimed to enroll children with JIA treated with methotrexate or biologic medications in any available formulations. This registry is named “Pharmacovigilance in JIA patients treated with biologic agents and/or MTX - Pharmachild” (European Union grant 260353) and is primarily aimed to evaluate the long-term safety of these therapeutic agents [56].

The findings of our study should be interpreted in the light of some potential caveats. Because our analysis was nonrandomized and observational, we cannot exclude that children still on treatment with ETN at the time of the study had a less severe disease than those discontinued from the medication or lost to follow-up. However, although the method used to assess the treatment outcome was different, the percentage of patients who were in clinical remission was similar between cross-sectional and retrospective populations. The disease status was only assessed at a single point of time, which precluded the possibility to establish whether clinical remission was sustained while receiving treatment or after its interruption, and how many patients were retreated. Adverse events were recorded through the retrospective review of patients’ clinical charts. A retrospective analysis is subject to missing and possibly erroneous data. Thus, some side effects may be underreported. Notably, owing to the non-prospective nature of data collection, we could not calculate the crude rates of adverse events per 100 person-years of exposure. Furthermore, we acknowledge that attribution of adverse events was not done. The main strengths of our study are related to the size of the cohort, the methodology used for case ascertainment which minimized potential selection bias, and the detailed procedures used to assess patients on ongoing ETN therapy.

## Conclusions

Our study shows that around half of children with non-systemic JIA achieve complete disease quiescence under treatment with ETN. The medication was overall well tolerated, as only one quarter of patients experienced clinically significant adverse events and less than 10% had treatment discontinued for toxicity.
